# Effects of malocclusion and orthodontic treatment on quality of life among orthodontic patients with craniofacial disorder compared to healthy controls: A cross-sectional study

**DOI:** 10.1007/s00056-024-00571-w

**Published:** 2025-02-03

**Authors:** C. Weismann, M. Schmidt, J. Effert, M. C. Schulz, C. F. Poets, B. Koos, M. Aretxabaleta

**Affiliations:** 1 Department of Orthodontics, University Hospital Tuebingen, Osianderstr. 2–8, 72076 Tuebingen, Germany; 2 Department of Oral and Maxillofacial Surgery, University Hospital Tuebingen, Osianderstr. 2–8, 72076 Tuebingen, Germany; 3 Department of Neonatology, University Hospital Tuebingen, Calwerstr. 7, 72076 Tuebingen, Germany; 4 Center for Cleft Lip, Palate and Craniofacial Malformations, University Hospital Tübingen, Osianderstr. 2–8, 72076 Tübingen, Germany

**Keywords:** Cleft lip and/or palate, Crossbite, Oral hygiene, Robin sequence, Oral health impact profile, Lippen-, Kiefer- und/oder Gaumenspalte, Kreuzbiss, Mundhygiene, Robin-Sequenz, Oral Health Impact Profile

## Abstract

**Purpose:**

Craniofacial disorders (CD) affect the Oral Health Impact Profile (OHIP). Therefore, this study evaluates the OHIP in orthodontic patients with cleft lip and/or palate or Robin sequence compared to healthy controls (C).

**Methods:**

A prospective, cross-sectional study was conducted. Oral health-related quality of life (OHRQoL) was assessed using the OHIP-14 questionnaire, with responses categorized into functional and psychological well-being items. In addition, the study considered the influence of crossbite, orthodontic appliance type, oral hygiene, and speech therapy. A high OHIP score represents a good quality of life. The Mann–Whitney test was used for nonparametric quantitative variables; statistical significance was set at *p* < 0.05.

**Results:**

The study included 119 participants (ages 7–21 years; 61 male, 58 female), divided into a CD group consisting of patients with cleft lip and/or palate or Robin sequence (*n* = 42) and a control group (C; *n* = 77; mean age 13.5 ± 5.2 and 14.3 ± 3.3 years, respectively). Both groups showed comparable OHIP-14 scores. The CD group reported significantly higher satisfaction regarding nutritional intake (*p* = 0.03), while the social and psychological dimensions were reduced (*p* = 0.04). Factors like crossbite, orthodontic appliance and speech therapy did not have a significant impact on OHIP. CD patients with good oral hygiene showed significantly superior self-reported psychological OHIP (*p* = 0.04).

**Conclusion:**

Patients with cleft lip and/or palate or with Robin sequence exhibited OHIP scores comparable to healthy individuals despite their underlying condition. Early guidance on dental care and tooth-friendly nutrition has the potential to improve OHRQoL. Additionally, providing supplemental psychological support during orthodontic treatment is advisable.

**Graphic abstract:**

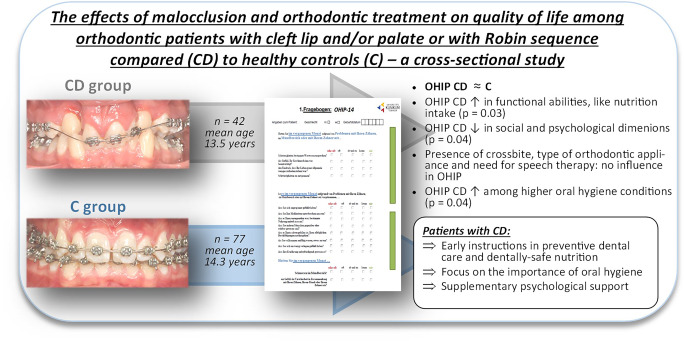

**Supplementary Information:**

The online version of this article (10.1007/s00056-024-00571-w) contains supplementary material, which is available to authorized users.

## Abstract (de)

**Zielsetzung:**

Kraniofaziale Anomalien (CD) beeinträchtigen das „Oral Health Impact Profile“ (OHIP). Daher wird in dieser Studie das OHIP bei kieferorthopädischen Patienten mit Lippen-, Kiefer- und/oder Gaumenspalte oder Robin-Sequenz im Vergleich zu einer gesunden Kontrollgruppe (C) untersucht.

**Methoden:**

Durchgeführt wurde eine prospektive Querschnittsstudie. Die mundgesundheitsbezogene Lebensqualität (OHRQoL) wurde anhand des OHIP-14-Fragebogens bewertet, bei welchem die Antworten in Kategorien für Aspekte des funktionellen und des psychischen Befindens eingeteilt sind. Darüber hinaus berücksichtigte die Studie den Einfluss von Kreuzbiss, Art der kieferorthopädischen Apparatur, Mundhygiene und Sprachtherapie. Ein hoher OHIP-Wert steht für eine gute Lebensqualität. Der Mann-Whitney-Test wurde für nichtparametrische quantitative Variablen verwendet, die statistische Signifikanz wurde auf *p* < 0,05 festgelegt.

**Ergebnisse:**

An der Studie nahmen 119 Probanden (7–21 Jahre; 61 männlich, 58 weiblich) teil, die in eine CD-Gruppe, bestehend aus Patienten mit Lippen-, Kiefer- und/oder Gaumenspalte bzw. Robin-Sequenz (*n* = 42), und einer gesunden Kontrollgruppe (C; *n* = 77; Durchschnittsalter 13,5 ± 5,2 bzw. 14,3 ± 3,3 Jahre) unterteilt wurden. Beide Gruppen wiesen vergleichbare OHIP-14-Werte auf. Die Gruppe mit CD berichtete über eine signifikant höhere Zufriedenheit in Bezug auf die Nahrungsaufnahme (*p* = 0,03), während die sozialen und psychologischen Dimensionen reduziert waren (*p* = 0,04). Faktoren wie Kreuzbiss, Art der kieferorthopädischen Apparatur und Sprachtherapie hatten keinen signifikanten Einfluss auf das OHIP. CD-Patienten mit guter Mundhygiene wiesen in der Selbsteinschätzung ein signifikant besseres psychologisches OHIP auf (*p* = 0,04) als Patienten mit schlechteren Hygienebedingungen.

**Schlussfolgerung:**

Patienten mit Lippen-, Kiefer- und/oder Gaumenspalte bzw. mit Robin-Sequenz wiesen trotz ihrer Grunderkrankung OHIP-Werte auf, die mit denen gesunder Probanden vergleichbar waren. Eine frühzeitige Anleitung zu Mundhygiene und zahnfreundlicher Ernährung trägt potenziell zur Verbesserung der OHRQoL bei. Darüber hinaus ist eine zusätzliche psychologische Unterstützung während der kieferorthopädischen Behandlung zu empfehlen.

## Introduction

The presence of a craniofacial disorder (CD) often necessitates more extensive orthodontic treatment [1]. Among CDs, cleft lip and/or palate (CL/P) are the most prevalent, occurring in about 1:600 births [2]. CL/P has a multifactorial etiology [3, 4] and cleft deformations result from a fusion deficiency of maxillary segments, manifesting as right- or left-sided unilateral cleft lip and palate (CLP), bilateral CLP, cleft lip with or without alveolus (CL± A) and isolated cleft palate (CP). Additionally, CP is found in 80–90% of patients with Robin sequence (RS), which has a prevalence of 1:8000 births [5]. The RS phenotype is characterized by mandibular retrognathia and glossoptosis, leading to upper airway obstruction and feeding problems [6]. Given the occurrence of a CD, special dentoskeletal growth patterns emerge. In RS, this involves a skeletal class II configuration due to bimaxillary retrognathia, a shorter ramus, and enlarged gonial angles [7–11], accompanied by the intraoral feature of dental crowding [12]. Extra-orally, RS patients exhibit a deficit in the lower facial proportions, resulting in a more convex profile as the soft tissue cannot mask the underlying CD [13]. In contrast, CL/P patients often show asymmetric growth and maxillary hypoplasia due to postoperative scar formation and maxillary cleft segmentation [14–16]. This disrupts and imbalances the stomatognathic system, resulting in significant physical and functional difficulties in daily life [17, 18], as well as in reduced oral hygiene that increases the risk of caries and periodontal disease [19]. Moreover, the presence of a CP often results in velopharyngeal insufficiency, adversely affecting speech development. Consequently, early speech therapy is crucial to support the multidisciplinary cleft care [20, 21].

Considering that CDs decrease physiological function, facial esthetics, and speech, patient dissatisfaction can be expected [22, 23], potentially leading to decreased social standing, negative self-perception and low self-esteem, as the face is vital for social interaction [24, 25]. If not properly addressed, these physiological effects may result in depression [26], behavioral issues [27], social stigmatization, bullying [28], reduced educational attainment [29], and exclusion [30]. Consequently, the Quality of Life (QoL) of patients with CD is likely to be compromised [31–34]. Therefore, comprehensive multidisciplinary treatment from birth to adulthood is required to achieve optimal physiologic function and psychological well-being.

The topic of QoL is gaining increasing attention in healthcare. In 1989, Reisine et al. first linked QoL with oral disease [35]. Since then, the term “oral health-related quality of life” (OHRQoL) has been developed to specifically address psychosocial and functional aspects of oral health [36, 37]. The Oral Health Impact Profile (OHIP) questionnaire is commonly used to examine OHRQoL [38–40]. It consists of 49 items (OHIP-49), but also has a shorter 14-item version (OHIP-14) [39, 40], which still offers high reliability, precision, and validity by covering each of the seven conceptual subgroups of the OHIP-49 [41].

Previous studies have assessed the OHRQoL in CL/P patients using questionnaires [42–44], such as the OHIP-14 [45–47]. Variability in methods, small sample size, and subgroup stratification led to inconsistent results, and study heterogeneity hampered comparisons across studies [48]. Currently, no research evaluating the influence of factors like the specific malocclusion, orthodontic treatment, speech therapy, and oral hygiene on OHRQoL exists. However, assessing these factors alongside rehabilitation could enhance our understanding and optimize therapy for better patient care.

Against this background, the current study aimed to evaluate OHRQoL in orthodontic patients with specific CDs and compare it with patients receiving orthodontic treatment but without any congenital disorder. Additionally, the impact of specific malocclusions, the employed orthodontic appliance type, speech therapy and oral hygiene level on the OHRQoL of the CD patients was assessed. The following null hypotheses were defined:

- The presence of a CD does not influence OHRQoL,
- Malocclusion does not affect the OHRQoL,
- Employed type of orthodontic appliance (fixed or removable) does not affect OHRQoL,
- Speech therapy does not affect the OHRQoL, and
- Oral hygiene level does not affect OHRQoL.

## Methods

The current study is part of a prospective, observational, cross-sectional study on the functional and psychological outcomes of orthodontic patients with and without CD. It is a follow-up to studies from Schmidt et al. [49] and Weismann et al. [50] reporting on the same sample and was approved by the institutional ethics committee of Tübingen University Hospital (registration number: 188/2019BO1). Written informed consent was obtained from caregivers, who were informed and invited to participate with their child during routine follow-up visits at the Department of Orthodontics in accordance with the principles of the Declaration of Helsinki.

### Participants

Detailed descriptions are available in previous reports [49, 50]. In brief, 119 participants aged 7–21 years were categorized into a CD or a healthy control (C) group. The CD group included orthodontic patients with nonsyndromic CD and all variations of CL/P, whereas the healthy controls consisted of children receiving orthodontic treatment, but without any congenital disorder. Patients with complex CD (e.g., syndromes), any other illness, or psychological limitations were excluded.

During an 8‑month recruitment period, a total of 140 children fulfilled the inclusion criteria. Of these, 21 were excluded due to poor compliance (e.g., missing appointments), restrictions imposed by the coronavirus disease 2019 (COVID-19) pandemic, or no interest in the study. The final sample consisted of 119 children, divided into 42 participants with CD and 77 control patients (C) (Table 1). The CD group included 4 RS and 38 CL/P patients. The mean age was 13.5 years (range 7–21 years) for the CD group and 14.3 years (range 7–21 years) in the C group, with an equal sex ratio for the C group and more males in the CD group. The response rate was 100%. For the comprehensive study concept the sample size was statistically defined with the predetermined ratio for the CD to the C group as two to one.

**Table 1:** Descriptive statistics according to age, sex, craniofacial disorder, cleft location, crossbite malocclusion, orthodontic appliance, speech therapy, and oral hygiene condition Deskriptive statistische Angaben nach Alter, Geschlecht, kraniofazialer Störung, Spaltlage, Kreuzbiss, kieferorthopädischer Apparatur, Sprachtherapie und Status der Mundhygiene

	CD group		Control group			CD group		Control group	
**Sex**	***n***	**%**	***n***	**%**	**Orthodontic appliance**	***n***	**%**	***n***	**%**
Male	25	59.5	36	46.8	Fixed	21	50.0	42	54.6
Female	17	40.5	41	53.3	Removable	21	50.0	35	45.5
**Craniofacial malformation**	***n***	**%**	***n***	**%**	**Oral hygiene**	***n***	**%**	***n***	**%**
RS	4	9.5	–		High	17	41.5	61	79.2
CL/P	32	76.2			Moderate	21	50.0	15	19.5
CP	4	9.5			Low	4	9.5	1	1.3
CL ± A	2	4.8			–				
**Cleft location**	***n***	**%**	***n***	**%**	**Speech therapy**	***n***	**%**	***n***	**%**
*Unilateral*	*26*	*81.3*	–		Yes	40	95.2	7	9.1
Left	18	56.3			No	2	4.8	70	90.9
Right	8	25.0			–				
*Bilateral*	*6*	*18.8*							
**Crossbite**	***n***	**%**	***n***	**%**	**Age**	**Mean**	**SD**	**Mean**	**SD**
Posterior	3	7.1	2	2.6	–	13.5	5.2	14.3	3.3
Anterior	8	19.1	2	2.6		–			
Circular	3	7.1	1	1.3					
None	28	66.7	72	93.5					

*CD* craniofacial disorder; *SD* standard deviation; *RS* Robin sequence; *CL/P* cleft lip and/or palate; *CP* cleft palate; *CL* *±* *A* cleft lip and/or alveolus

### Oral health impact profile-14 questionnaire

The German version of the OHIP-14, consisting of 14 items, was used for a patient self-report measure of his/her OHRQoL [41]. Answers were separated into functional and psychological well-being items, with a 5-point Likert scale from “never” (0) to “very often” (4) for frequency of negative or positive experiences over the past 3 months. Resulting values were added, with higher scores indicating better oral health. Values were aggregated by subgroup and overall. The study framework was integrated into clinical practice to foster clinician–patient engagement, ensuring accurate survey completion and query clarification. Surveys were conducted face-to-face, with the orthodontist acting as an interviewer chairside. Importantly, the interviewer refrained from influencing answers, only providing clarification in unclear questions. This meticulous standardization aimed at establishing uniform conditions, reducing the impact of age-related variations and minimize bias during independent interviews.

### Orthodontic and dental therapy parameters

During follow-up visits, orthodontic and dental clinical examinations were performed by the same orthodontist, employing the same instruments. These measurements were part of the medical record at the Department of Orthodontics. Intraoral assessments of dental malocclusion, including the transversal relationship of the anterior and posterior maxillary and mandibular arch, were performed chairside to detect any crossbite (anterior, posterior, circular, or none). Oral hygiene status was visually evaluated and classified as high (no plaque or discoloration), moderate (localized plaque and/or discoloration), or low (generalized plaque and discoloration). Moreover, information on speech therapy attendance (yes/no) and the employed type of orthodontic appliance (fixed/removable) was obtained.

### Statistical data analyses

Patient data collected from the electronic database, clinical records, and pseudonymized forms were exported in an Excel sheet (Microsoft® Inc., Redmond, WA, USA). Statistical analyses were performed using SPSS® Statistics (Version 29, IBM, Armonk, NY, USA). Descriptive statistics included means and standard deviations (SD) or median and range, as appropriate. The Shapiro–Wilk test assessed data distribution. The Mann–Whitney test was applied to evaluate possible significant differences between both patient groups in the mean score results. Additionally, means, SDs, medians, ranges, and floor-to-ceiling effects were calculated for the various questionnaire items. Scores were evaluated based on the type of the employed orthodontic appliance, speech therapy, oral hygiene condition, and presence of crossbite malocclusion. Statistical significance was set to 5% (*p* = 0.05).

## Results

### Characteristics of participants

The most common CD was CL/P, with a left unilateral CLP being twice as frequent as a right unilateral or bilateral one (Table 1). There were equal numbers of subjects with isolated CP and RS, but fewer with CL ± A. Crossbite malocclusions were more frequent in the CD group, where the anterior variant was predominant, followed by the posterior and circular one, which were equally common. Due to the low sample size, all crossbite types were grouped together for further evaluation. The different types of orthodontic appliances were equally distributed across the CD patients, while fixed appliances were slightly more frequently applied in the C group. Nearly all CD patients attended speech therapy. In contrast, very few C participants underwent speech therapy. Finally, half the patients with CD demonstrated moderate oral hygiene, while most of the C group presented with a high hygiene level.

### Oral health impact profile-14 questionnaire

The OHIP-14 questionnaire results showed a high QoL in both groups (Table 2, Supplementary Fig. 1). Scores for both subgroups, functional and psychological well-being, as well as for the total score, showed comparable results for both groups. Comparable results were also found for individual questions, except for questions 5 (“nutrition unsatisfactory”) and 10 (“tended to be irritable”). For question 5, participants with CD were significantly more satisfied with their nutrition performance than the healthy controls (*p* = 0.03), with the CD patients exclusively selecting the highest positive value (4, “very often”). In contrast, healthy participants indicated lower scores for the same question, with some outliers ranging from “occasionally” (2) to “very often” (4). For question 10, the CD group reported a significantly decreased QoL compared to the C group (*p* = 0.03).

**Table 2:** Oral Health Impact Profile-14 (OHIP-14) comparing patients with craniofacial disorder (CD; *n* = 42) and controls (C; *n* = 77), where both subscores (functional and psychological well-being) and total scores are compiled Oral Health Impact Profile-14 (OHIP-14) zum Vergleich von Patienten mit kraniofazialer Störung (CD; *n* = 42) und Personen der Kontrollgruppe (C; *n* = 77), bei dem sowohl die Teilwerte (funktionelles und psychologisches Wohlbefinden) als auch die Gesamtwerte erfasst werden

Subscores and items	Control group							Craniofacial disorder group							
	Score			Floor to ceiling effect				Score			Floor to ceiling effect				Sig.
	Mean (SD)	Median	Range	% of value 0	% of value 4	S	K	Mean (SD)	Median	Range	% of value 0	% of value 4	S	K	
**Functional well-being**															
1. Difficulty saying certain words	3.5 (1.0)	4	(0–4)	2.6	70.1	−2	3.5	3.2 (1.4)	4	(0–4)	9.5	71.4	−1.5	0.7	0.73
2. Altered taste perception	3.9 (0.4)	4	(1–4)	0	96.1	−5.9	36.1	4.0 (0.2)	4	(3–4)	0	95.2	−4.4	18.3	0.85
3. Necessity of meal interruption	3.8 (0.7)	4	(1–4)	0	85.7	−3.0	9.0	3.8 (0.6)	4	(2–4)	0	88.1	−2.8	6.9	0.73
4. Discomfort with specific foods	3.4 (1.0)	4	(0–4)	2.6	71.4	−1.8	2.3	3.6 (0.9)	4	(0–4)	2.4	76.2	−2.3	4.8	0.54
5. Unsatisfactory nutrition	3.9 (0.5)	4	(2–4)	0	89.6	−3.3	10.2	4.0 (0.0)	4	(4–4)	0	100.0	–	–	0.03*
6. Pain in the mouth area	3.2 (1.1)	4	(0–4)	2.6	57.1	−1.3	1.0	3.2 (0.9)	3	(1–4)	0	47.6	−0.7	−0.5	0.58
*Score—Functional well-being*	21.7 (2.9)	22	(8–24)	0	35.1	−2.1	6.4	21.8 (2.6)	22	(13–24)	0	28.6	−1.7	3.0	0.93
**Psychological well-being**															
1. Overall life dissatisfaction	3.7 (0.8)	4	(1–4)	0	84.4	−2.5	5.5	3.7 (0.8)	4	(0–4)	2.4	81.0	−3.3	12.2	0.71
2. Difficulties to relax	3.4 (0.9)	4	(1–4)	0	67.5	−1.5	1.1	3.6 (0.7)	4	(1–4)	0	73.8	−2.0	3.6	0.39
3. Felt tense	3.5 (0.9)	4	(0–4)	1.3	71.4	−1.7	2.3	3.7 (0.8)	4	(0–4)	2.4	78.6	−3.1	11.4	0.31
4. Proneness to irritability	3.8 (0.5)	4	(2–4)	0	89.6	−3.0	7.8	3.6 (0.9)	4	(0–4)	2.4	73.8	−2.4	6.5	0.03*
5. Difficulties carrying everyday activities	3.9 (0.4)	4	(2–4)	0	90.9	−3.6	13.1	3.9 (0.6)	4	(0–4)	0	97.6	−6.5	42.0	0.18
6. Completely unable to do anything	4.0 (0.2)	4	(3–4)	0	97.4	−6.1	35.9	3.9 (0.7)	4	(0–4)	2.4	95.2	−5.1	27.0	0.51
7. Felt a little embarrassed	3.8 (0.6)	4	(0–4)	1.3	90.9	−4.1	18.3	3.6 (1.0)	4	(0–4)	2.4	81.0	−2.3	4.7	0.11
8. Teeth, mouth, or denture insecurity	3.7 (0.7)	4	(1–4)	0	83.1	−2.7	6.7	3.7 (0.9)	4	(0–4)	2.4	85.7	−3.1	9.4	0.79
*Score—Psychological well-being*	29.9 (3.3)	32	(12–32)	0	50.6	−2.6	10.1	29.6 (4.1)	30.5	(11–32)	0	40.5	−3.3	12.2	0.61
*Total score*	51.5 (5.9)	53	(20–56)	0	28.6	−2.6	10.3	51.3 (5.8)	53	(30–56)	0	19.0	−2.5	6.7	0.58

Mean, standard deviation (SD), median, range, floor-to-ceiling effect, kurtosis (S), and skewness (K) for each of the evaluated items were calculated for both patient groups. Values were compared by the Mann–Whitney test. Statistical significance (Sig.) was considered as *p* < 0.05 and denoted with *

### Influence of orthodontic and dental therapy parameters

**Fig. 1:**
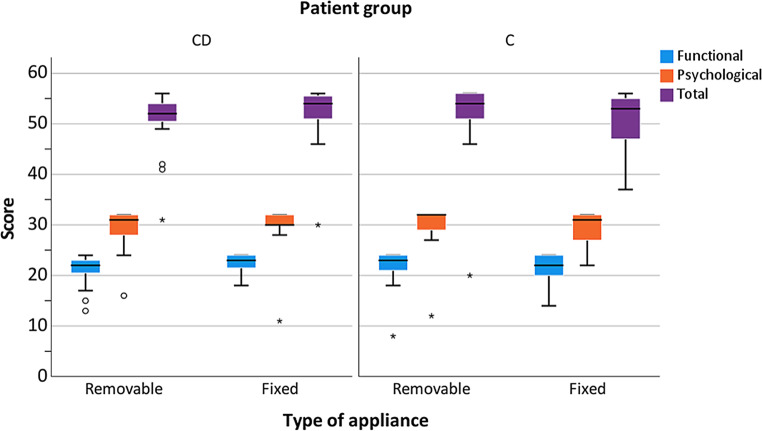
Oral Health Impact Profile-14 (OHIP-14) comparing patients with craniofacial disorder (CD; *n* = 42) and controls (C; *n* = 77), considering the employed orthodontic appliance (removable or fixed) for the evaluation of the values for the subscores (functional and psychological well-being) and total scores. *Data outlier Oral Health Impact Profile-14 (OHIP-14) im Vergleich von Patienten mit einer kraniofazialen Anomalie (CD; *n* = 42) zu einer gesunden Kontrollgruppe (C; *n* = 77) unter Berücksichtigung der verwendeten kieferorthopädischen Apparatur bei der Erhebung der Werte für die Teilscores (funktionelles und psychologisches Wohlbefinden) und des Gesamtscores. *Datenausreißer

**Fig. 2:**
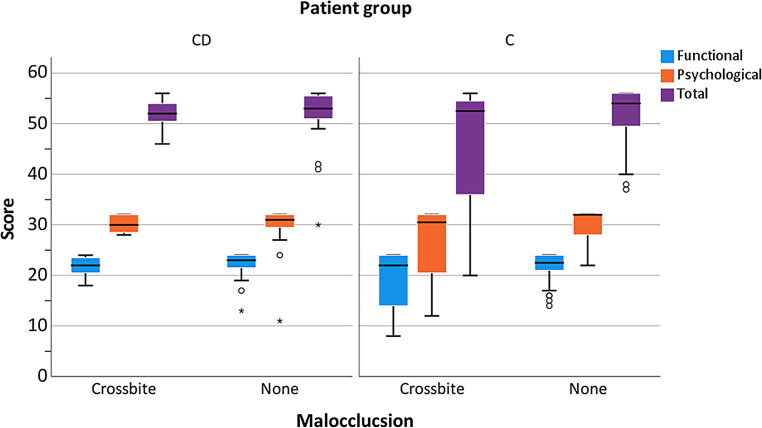
Oral Health Impact Profile-14 (OHIP-14) comparing patients with craniofacial disorder (CD; *n* = 42) and controls (C; *n* = 77), considering the occurrence of a crossbite for the evaluation of the values for the subscores (functional and psychological well-being) and total scores. *Data outlier Oral Health Impact Profile-14 (OHIP-14) im Vergleich von Patienten mit einer kraniofazialen Anomalie (CD; *n* = 42) zu einer gesunden Kontrollgruppe (C; *n* = 77), unter Berücksichtigung des Vorliegens eines Kreuzbisses bei der Erhebung der Werte für die Teilscores (funktionelles und psychologisches Wohlbefinden) und des Gesamtscores. *Datenausreißer

**Fig. 3:**
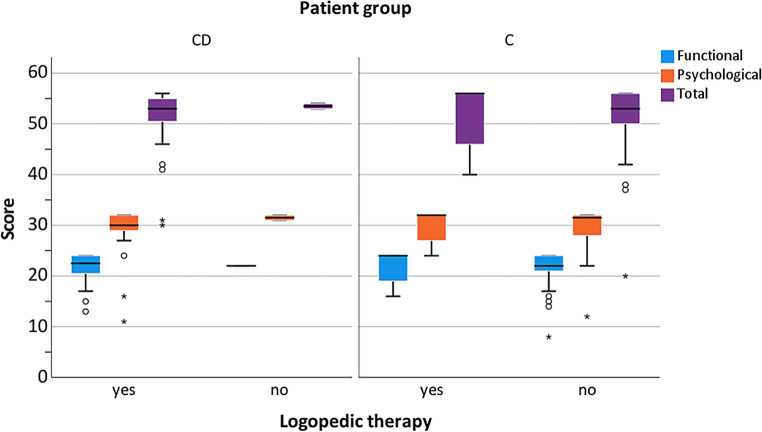
Oral Health Impact Profile-14 (OHIP-14) comparing patients with craniofacial disorder (CD; *n* = 42) and controls (C; *n* = 77), considering the attendance to speech (logopedic) therapy for the evaluation of the values for the subscores (functional and psychological well-being). *Data outlier Oral Health Impact Profile-14 (OHIP-14) im Vergleich von Patienten mit einer kraniofazialen Anomalie (CD; *n* = 42) zu einer gesunden Kontrollgruppe (C; *n* = 77), unter Berücksichtigung einer Sprachtherapie (Logopädie) bei der Erhebung der Werte für die Teilscores (funktionelles und psychologisches Wohlbefinden) und des Gesamtscores. *Datenausreißer

**Fig. 4:**
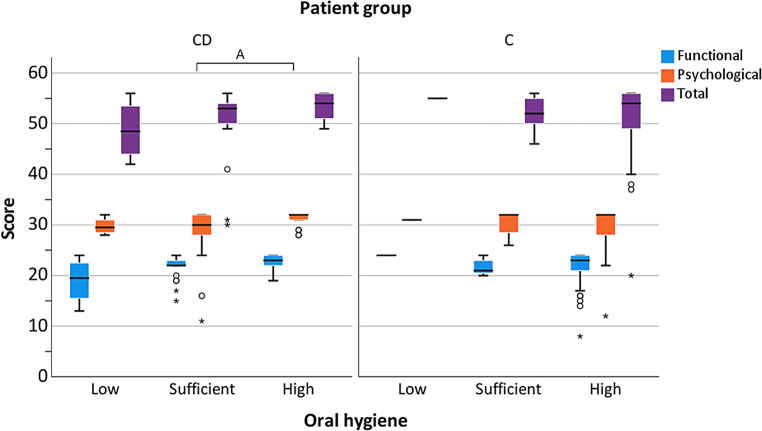
Oral Health Impact Profile-14 (OHIP-14) comparing patients with craniofacial disorders (CD; *n* = 42) and controls (C; *n* = 77), considering the level of oral hygiene (low, sufficient, high) for the evaluation of the values for the subscores (functional and psychological well-being) and total scores. Values were compared by the Mann–Whitney test. Statistical significance was considered at *p* < 0.05 and denoted with (A). *Data outlier Oral Health Impact Profile-14 (OHIP-14) im Vergleich von Patienten mit einer kraniofazialer Störung (CD; *n* = 42) zu einer gesunden Kontrollgruppe (C; *n* = 77), unter Berücksichtigung des Niveaus der Mundhygiene (gering, ausreichend, hoch) für die Erhebung der Werte für die Unterscores (funktionelles und psychologisches Wohlbefinden) und des Gesamtscores. Die Werte wurden mit dem Mann-Whitney-Test verglichen. Eine statistische Signifikanz wurde bei *p* < 0,05 angenommen und mit (A) gekennzeichnet. *Datenausreißer

Regarding the appliance type, no significant differences were found (Fig. 1). Standard deviations were found to be higher in the C than in the CD group, especially for the total score (Fig. 1). Concerning the occurrence of crossbite, also no significant group differences were found (Fig. 2). Comparable results were obtained regarding the subscores (functional *p* = 0.66, psychological *p* = 0.66) as well as for the total score (*p* = 0.66). Regarding the C group, for all analyzed subscores and crossbite groups, larger standard deviations were found compared to the CD group. Regarding attendance to speech therapy, there was no significant effect on the OHIP-14 total score or any subscore (Fig. 3). Whereas almost all CD patients attended speech therapy, almost none of the C group did this. This means that the most reliable calculations were those for attendance in the CD (40 of 42 patients) and no attendance in the C group (70 of 77 patients). Referring to the effect of oral hygiene on QoL, better hygiene was associated with higher QoL (Fig. 4). No significant difference in the extent of oral hygiene between them (*p* = 0.72).

Few patients from the CD group (*n* = 4) and only one from the C group were in the lowest category for oral hygiene, thus, precluding statistical analysis. In the CD group, patients with a high oral hygiene level showed significantly higher results for the psychological subscores compared to those with sufficient hygiene (*p* = 0.04).

## Discussion

In this study, the OHRQoL of patients with cleft lip and/or palate or with Robin sequence (CD patients) undergoing orthodontic treatment was evaluated using the OHIP-14 questionnaire and compared to a control group. In addition, the effect of the type of the employed orthodontic appliance, the presence of a crossbite, speech therapy, and the level of oral hygiene on the OHIP were evaluated.

OHRQoL may be regarded as a broad concept, covering aspects of functional and psychological well-being during everyday life and the oral health of an individual [51]. Therefore, the current study divided the questions of the OHIP-14 into subgroups focusing on functional and psychological well-being. We could show that orthodontic patients with CD had an OHRQoL comparable to that of healthy controls. Thus, our null hypothesis that the CDs will not affect OHRQoL was accepted. Corcoran et al. studied 63 patients with CL/P (mean age 18 years) at the date of their final follow-up visit also using the OHIP-14 questionnaire [45]. They found that the underlying CD had a negative impact on OHRQoL in more than half their patients, contrary to the current study. They also reported that psychological discomfort and physical pain were more predominant in CL/P patients, which aligns with our observation that irritability was more frequent in patients with CD. However, no negative impact on OHRQoL due to functional aspects was observed. Participants reported equal or even higher satisfaction compared to healthy individuals. However, a reduction in OHRQoL was noted among CD patients, primarily attributed to psychological factors. Compared to the study by Corcoran et al., our sample consisted of younger patients and followed a different treatment concept. Moreover, the validity of the questionnaires can vary across languages and cultures, as was shown comparing studies performed in Finland and Germany [52], which might have introduced bias. Overall, our results suggest that CD patients can cope with their condition, although some differences in psychological and social subdomains remain. This is supported by other studies [44, 46, 53, 54], which suggests that social and psychological problems are interconnected and can persist throughout the rehabilitation process. The relationship between social stereotyping and appearance is well-documented in social sciences [55–57]. Unlike some other health problems, CD is a visible condition that affects facial appearance, reducing esthetic attractiveness [58]. This stigmatization significantly impacts on the patients’ psychological well-being but also on their development and social life. Early identification of these issues during the interdisciplinary treatment can help address this [33], providing reassurance to patients during their development. In this regard, it is encouraging to note that CD patients generally adapt well to life despite their condition [59].

CDs are commonly associated with poor oral hygiene and the latter was also shown to be linked to the extent of the tissue defect [19, 60]. The current results showed a significant association between the psychological well-being subscore and oral hygiene of the CD group. A similar trend was observed in the C group, albeit without statistical significance. Therefore, the proposed hypothesis that oral hygiene does not affect OHRQoL was accepted for the CD patients and rejected for the C group. Pasini et al. assessed OHRQoL by using an Italian version of the OHIP-14 questionnaire in CL/P patients who had attended a tailored oral hygiene program from an early age [31]. They compared the CL/P patients (*n* = 32, mean age 9.8 years) to healthy participants (*n* = 32, 10.1 years) and found that the patients with CD had significantly lower mean OHIP-14 scores than the healthy controls, despite the oral hygiene program. This aligns with our data, as a higher number of CD patients demonstrated sufficient rather than high oral hygiene (Table 1). Although the overall OHIP score was not significantly affected, the psychological well-being score was significantly influenced. This confirms the importance of an early preventive dental health care program and the education on tooth-friendly nutrition to improve oral hygiene.

For gathering information on the stomatognathic system affecting OHRQoL, the occurrence of malocclusion, specifically of crossbite, was evaluated concerning the OHIP results. Previous studies from our group evaluating the same patient population identified the presence of a crossbite as significantly impacting mastication efficiency [49, 50]. Overall, orthodontic patients without a crossbite did not show significantly different OHIP scores, so the related null hypothesis was confirmed. Some other studies suggested that the presence of a malocclusion deteriorated patients’ QoL [32, 61–64], but here CD patients were not considered. Moreover, crossbite, particularly in the posterior dental arch, may induce asymmetric growth, which not only may affect QoL due to reduced esthetics [65], but may also cause functional problems leading to temporomandibular symptoms [66]. Also, the severity of the manifested malocclusion was linearly correlated to negative emotional, psychosocial, and psychological effects [67]. Esthetic appearance, in this case the abnormal facial appearance manifested by the CD, was associated with bullying [68], which may result in social withdrawal and low self-esteem [69, 70].

Orthodontic malocclusion treatment typically begins during adolescence but may start earlier in CD patients due to maxillary growth restrictions. Adolescence is a critical period when individuals are more likely to reflect and compare themselves with peers, becoming more aware of their social life and appearance. Therefore, addressing orthodontic problems earlier can greatly benefit patients with a CD, reducing the impact of the malocclusion and its effect on OHRQoL. Additionally, early intervention helps them adapt to their reduced esthetic appearance. This benefit was proven by Vaiciunaite et al., who reported that up to 65% of CL/P patients reported higher confidence and happiness after starting orthodontic treatment [71].

During early rehabilitation treatment, the implementation of speech therapy, similar to that performed in our facility, is highly recommended [72–74]. Further studies are needed regarding the effects of early speech therapy, particularly in preschool children [74–76]. It is imperative that speech rehabilitation be started well in advance of starting school to reduce the risk of stigmatization. With increasing age, differences between individuals with CD and healthy controls decrease [77]. In our CD group, almost all patients had agreed to attend speech therapy (Fig. 4), but its impact on the overall OHRQoL could not be identified. Therefore, the null hypothesis stating that speech therapy did not affect the OHRQoL was accepted.

Few studies have investigated the impact of orthodontic therapy on the OHRQoL [32, 78], particularly considering patients suffering from CD or looking at the specific type of removable appliance used. The present study revealed a role of these factors, although, because of methodological heterogeneity, comparison with other studies is difficult [78]. The following claims only refer to otherwise healthy orthodontic patients who were treated with fixed appliances. The literature indicates that OHRQoL was higher before treatment than in the initial weeks after incorporating the appliance. During this period, OHRQoL decreased in terms of functional well-being but increased in emotional and social welfare subdomains [79–82], a trend confirmed by parents and caregivers as well [83]. The scores improved as orthodontic treatment progressed, with the best results seen after its completion [78, 84]. The previously referenced literature typically assessed only a single patient group, which may not be directly comparable with the current study’s population. Furthermore, participants in the current study had been undergoing orthodontic treatment for a longer period, making them accustomed to their appliances. Thus, any OHRQoL deficit induced by the CD may have been compensated once subjects became more familiar with their condition. Sergl et al. studied the social and functional discomfort of orthodontic patients with removable and fixed appliances (*n* = 48; mean age 13 years) during the first 7 days of therapy and again after 0.5, 3, and 6 months [85]. They found that the highest negative impact occurred directly after appliance incorporation, but decreased significantly during the first 7 days, thus, having no long-term effect. Therefore, no significant differences in the impact on the OHRQoL were found concerning the type of orthodontic appliance used, confirming the related hypothesis.

The current study has several limitations. First, the limited prevalence of CD, the monocentric study design, specific inclusion criteria (e.g., same orthodontic treatment approach from the cleft center), and disruptions caused by the COVID-19 pandemic (affecting appointment regularity), led to a small sample size. To mitigate this problem, the CD group encompassed different types of CDs to increase sample size. This may have introduced bias, as RS has a unique skeletal growth pattern and different functional problems compared to isolated CL/P patients, potentially influencing the OHRQoL. Second, the study encompassed a broad age range. Unfortunately, the inherent constraints of a low prevalence and a monocentric study design rendered this unavoidable. The patients’ age range was expanded not only to increase the sample size but also to capture various treatment stages, as well as individuals’ psychological and physiological development. We acknowledge the diverse life stages of our participants and their impact on well-being; however, this variability is also inherent to the subjective nature of self-reported surveys. Moreover, conducting the survey in an interviewer–patient setup for all participants minimized the risk of misinterpretation of questions and potential challenges in survey completion, especially among younger participants. The consistent application of our protocol for all participants within one cleft center is pivotal for mitigating bias. Despite these limitations, the obtained results are both encouraging and valuable for comprehending self-reported differences between the two study groups within one center.

There is a compelling need for future research. Particularly, there is a gap in literature regarding OHRQoL in CD patients undergoing current orthodontic treatment. Future studies should include an age-matched control group without orthodontic treatment to better understand the detailed effects on QoL. Additionally, a longitudinal study design, assessing the questionnaire before and after orthodontic treatment, would allow further insights into the specific causes affecting QoL and their impact on outcomes. Long-term, these findings should enhance patient care.

## Conclusion

Within the limitations of this study, the following can be concluded:

- Patients with the specific craniofacial disorders (CD) cleft lip and/or palate or Robin sequence demonstrated an oral health-related quality of life (OHRQoL) comparable to that observed in healthy controls, suggesting potential benefits from their orthodontic treatment.
- The patients with CD exhibited significantly higher satisfaction with their functional abilities, like nutritional intake and ingestion, but experienced a significant decrease in OHRQoL in social and psychological dimensions.
- The presence of crossbite, type of orthodontic appliance employed, and adherence to speech therapy showed no significant influence on the OHRQoL.
- Oral hygiene levels were found to impact the OHRQoL, with higher oral hygiene correlating with elevated psychological well-being subscores.

Early preventive guidance in dental care and nutrition, aimed at both, patients with CD and their caregivers, can significantly impact OHRQoL. Moreover, promptly identifying and addressing psychological and social challenges should be a priority in the interdisciplinary treatment of patients with CD. This focus is crucial not only for preventing the escalation of issues but also for providing patients a sense of security and comfort. These considerations contribute significantly to sustaining motivation, fostering discipline and ensuring compliance throughout the treatment process.

## Supplementary Information

Supplementary Fig. 1

## Data Availability

Data will be made available on reasonable request.
