# Supplementary material for: Effects of malocclusion and orthodontic treatment on quality of life among orthodontic patients with craniofacial disorder compared to healthy controls: A cross-sectional study
Source: J Orofac Orthop. 2025 Feb 3;87(3):239–52. doi: 10.1007/s00056-024-00571-w (PMC13109231; doi:10.1007/s00056-024-00571-w)
Supplement: Supplementary file 1 — Supplementary Fig. 1 [file 56_2024_571_MOESM1_ESM.pdf]

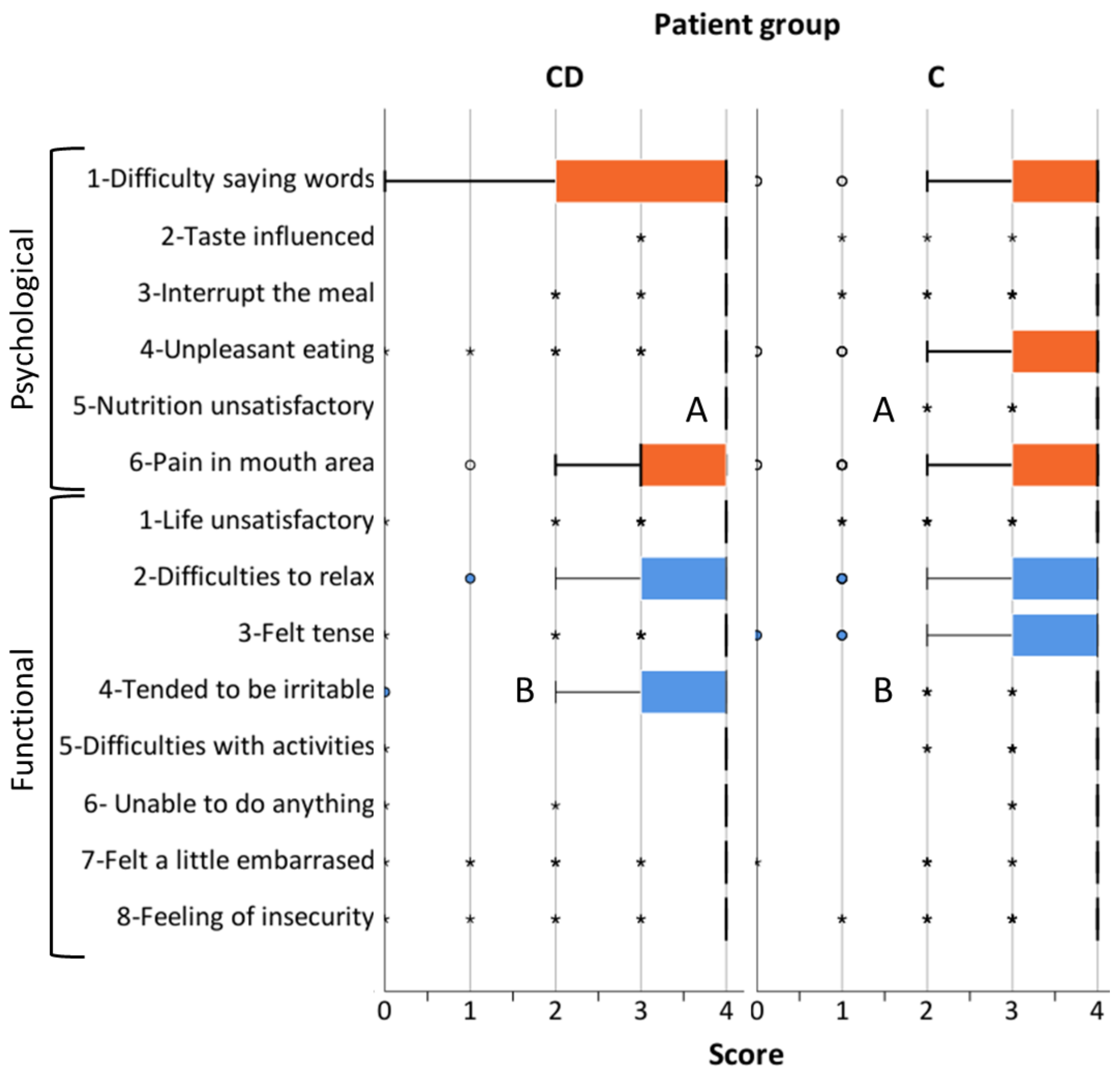

Supplementary Figure 1: Visualization of the Oral Health Impact Profile-14 (OHIP-14) comparing patients with craniofacial disorder (CD) (n = 42) and controls (C) (n = 77) without CD considering the median OHIP-14 scores and subscores (functional [blue] and psychological [orange] well-being). OHIP-14 scores of the different groups were compared by the Mann-Whitney test. Statistical significance was considered at $p < .05$ and denoted with (A and B). Data outliers are given by stars
